# Effect of *in situ* aspartame mouthwash to prevent intrinsic and extrinsic erosive tooth wear

**DOI:** 10.4317/jced.56857

**Published:** 2020-07-01

**Authors:** Franciny-Querobim Ionta, Marcela-de Azevedo-Garcia Bassoto, Natália-Mello dos Santos, Fabiana Di Campli, Heitor-Marques Honório, Thiago Cruvinel, Marilia-Afonso-Rabelo Buzalaf, Daniela Rios

**Affiliations:** 1 DDS, PhD; Assistant Professor, Department of Odontology. University of Marília; 2 DDS, Graduate student, Department of Pediatric Dentistry, Orthodontics and Public Health Bauru. Bauru School of Dentistry, University of São Paulo. Brazil; 3 DDS, PhD student, Department of Pediatric Dentistry, Orthodontics and Public Health Bauru. Bauru School of Dentistry, University of São Paulo. Brazil; 4 DDS, MSc student, Department of Pediatric Dentistry, Orthodontics and Public Health. Bauru School of Dentistry, University of São Paulo. Brazil; 5 DDS, PhD; Associate Professor, Department of Pediatric Dentistry, Orthodontics and Public Health. Bauru School of Dentistry, University of São Paulo. Brazil; 6 DDS, PhD; Full Professor, Department of Biological Sciences. Bauru School of Dentistry, University of São Paulo. Brazil

## Abstract

**Background:**

The aim was to evaluate whether aspartame regular mouthwash prior to erosive challenges with citric or hydrochloric acids would be able to prevent erosive enamel wear.

**Material and Methods:**

This randomized, single blind *in situ* study was conducted with 3 crossover phases of 5 days. Polished bovine enamel blocks (n=252) were randomly divided among 6 groups/ 3 phases/ 21 volunteers. The groups under study were: aspartame solution (0.024% of aspartame in deionized water - experimental group), deionized water (negative-control) and stannous-containing solution (Elmex® Erosion Protection Dental Rinse; positive-control); subjected to erosion on citric acid or hydrochloric acid. Four times per day the volunteers rinsed the intraoral appliance with the respective solutions (*in situ*) prior to immersion of half of the appliance in 0.05M citric acid and the other half in 0.01M hydrochloric acid for 120 seconds (extraoral). The response variable was enamel loss by profilometry. Data were analyzed by ANOVA and Tukey’s test (*p*<0.05).

**Results:**

No difference on enamel loss was found between aspartame solution and deionized water. Stannous-solution resulted in less enamel loss compared to deionized water. Hydrochloric acid resulted in higher enamel loss than citric acid.

**Conclusions:**

In this model, aspartame was not able to prevent erosive tooth wear against citric or hydrochloric acids.

** Key words:**Dental erosion, aspartame, stannous fluoride, citric acid, hydrochloric acid.

## Introduction

Dental erosion can be defined as the softening of dental surface due to the exposure of dental hard tissue to extrinsic or intrinsic acids of non-bacterial origin (1). Whether the acid exposure is prolonged or even under physiological condition with the incidence of mechanical forces by tooth attrition or abrasion of the tongue, this vulnerable softened layer can be removed causing tooth hard substance loss denominated erosive tooth wear (1,2). At routine examination, erosive tooth wear becomes clinically visible in later stages, when the appearance and shape of the teeth are compromised. In patients with gastroesophageal reflux or with eating disorders such as bulimia, stomach acids can be constantly present in the oral cavity leading to severe erosive wear with extensive loss of enamel and dentine (3). The risk of development of erosive tooth wear is also related to nutritional habits, such as high consumption of soft drinks and specific diets with increased consumption of acid fruits (4). The high prevalence of this condition (5-7) and the impact on patient’s quality of life is a cause of concern to dental clinicians and researchers.

Due to the high consumption of soft drinks, researches have been conducted regarding the erosive effects of different versions of soft drinks (8). Previous studies had found that light cola, which contains aspartame and a slight increase in the pH, presents less erosive potential compared to its traditional version (9,10). It was speculated that the presence of the amino acid phenylalanine, which is provided from the hydrolysis of aspartame in the presence of saliva could be the responsible for the lower erosive potential of the light cola drink (9,10). However, these studies were only conducted *in vitro*, without considering what really occurs in the mouth, regarding the presence of natural saliva and episodes of abrasion by the tongue, for example. Also, the anti-erosive potential of aspartame, regardless of its association with cola drinks, has not been previously evaluated. Thus, the aim of this *in situ/ex vivo* study was to evaluate whether aspartame regular mouthwash prior to immersion in citric or hydrochloric acids would be able to prevent erosive tooth wear when compared to deionized water and stannous-containing solution. The null hypothesis tested was that there is no difference among the tested solutions regarding their preventive effect against both kinds of acid challenges.

## Material and Methods

-Study design

This experiment followed a single-blind (for research), placebo-controlled, randomized, 3 *in situ/ex vivo* crossover phases in which the independent variables were type of treatment (in three experimental levels) and type of challenge (in two experimental levels). A washout period of 7 days was established between each phase. Polished bovine enamel blocks (n=252) were random divided among groups (n=6) and volunteers (n=21). The groups under study were: 0.024% of aspartame in deionized water (experimental groups), deionized water (negative-control) and stannous (Sn)- containing solution (Elmex® Erosion Protection Dental Rinse; positive-control); half of the enamel blocks was subjected to erosion in citric acid and the other half in hydrochloric acid (both Sigma Alderich - Merck, Darmstadt, Germany). The dependent variable was enamel loss (in µm) quantified profilometrically.

-Enamel blocks preparation

Enamel blocks (4 x 4 x 3 mm) were prepared from the labial surfaces of bovine incisors crowns using a cut device (ISOMET Low Speed Saw, Buehler Ltd., Lake Bluff, IL, USA) and double-sided diamond discs (XL 12205, “High concentration”, 102 x 0,3 x 12,7 mm3 Extec Corp., Enfield, CT, USA/ Ref: 12205). The blocks’ surfaces were ground flat with water-cooled silicon carbide discs (320, 600, and 1200 grade papers; Buehler, Lake Bluff, IL, USA), and polished with felt paper wet by 1 µm diamond spray (Buehler, Ltd., Lake Bluff, IL, USA). Two hundred and fifty-two blocks were selected and randomized among 8 studied groups and the 16 volunteers according to their surface hardness (Hardness tester from Buehler, Lake Bluff, IL, USA; five indentations in each block using Knoop diamond with 25 g for 10 seconds; enamel mean surface hardness of 341.20 ± 33 KPa/mm2). Before the *in situ* phase, the blocks were sterilized by ethylene oxide gas exposure.

-Baseline profilometric analysis

The surface of the enamel blocks were marked with a scalpel blade (Embramac, Itapira, SP, Brazil) for definition of the reference areas of 1.0 mm (at the border) and test area of 2.0 mm (at the center). Subsequently, five baseline surface profiles were obtained from the blocks using a profilometer (MarSurf GD 25, Göttingen, Germany) and a contour software (MarSurf XCR20). Blocks were fixed to a special holder to standardize the position and the location was recorded allowing their exact repositioning after the *in situ* phase. The surface profiles (3.0 mm in length) were obtained at the following distances of relative position of the block on the y-axis: 0.5, 0.75, 1.0, 1.25 and 1.5 μm. After the initial profilometry, the reference areas (at the border of enamel surface) were covered with nail varnish (Maybelline Colorama, Brazil), to be used as a reference for intact enamel during measurements of enamel loss.

-Volunteers and *in situ/ex vivo* phase

This study was conducted according to the guidelines of good clinical practice and conformed to the Declaration of Helsinki. The study has been independently reviewed and approved the local institutional ethics committee (CAAE nº 48729115.0.0000.5417). Informed consent was obtained from all individual participants included in the study. A sample of nineteen healthy volunteers was required considering a minimally detectable difference of 0.35 μm of enamel loss and 0.34 μm of standard deviation obtained in a pilot study with 3 volunteers, an 5% α error, and 20% β error was adopted. Two extra volunteers were added to account for possible drop out. Twenty-one healthy adult volunteers (dental students and graduate students of the local institution, aged 18–35 years) participated in the study. The inclusion criteria were residing in the same fluoridated area (0.70 mg F/L), stimulated salivary flow rate >1 mL/min, non-stimulated salivary flow rate >0.25 mL/min, and adequate oral health with no caries, erosion lesions and gingivitis/periodontitis. The exclusion criteria were systemic illness, gastroesophageal reflux, pregnant or breastfeeding women, current orthodontic intervention, professional application of highly concentrated fluoride compounds in the last 2 months, smokers, and users of acidic medications.

Before starting the *in situ* phase, volunteers were properly trained on how to perform the protocol. They received the study material and written instructions. Seven days prior to and during the experimental phase, the volunteers brushed their teeth after meals with a standard toothbrush (Curaprox 5460 ultra-soft, Curaden Swiss, Switzerland) and fluoride toothpaste (Triple Action, 1.450 ppm F, Colgate, Brazil) without the intraoral appliance. In addition, volunteers were informed not to use any other fluoride product.

Three acrylic resin removal palatal appliances were made for each volunteer to be used in each phase of the study. The appliances had four 6 × 4 × 3 mm cavities, two on each side, for block fixation. The blocks were fixed with wax (Asfer, Asfer Indústria Química, São Caetano do Sul, SP, Brazil) and adjusted to the level of the appliance surface. In one side of the appliance the enamel blocks were fixed with green wax (citric acid) and on the other side with blue wax (hydrochloric acid) to identify which blocks should be immersed in citric or hydrochloric acids. Volunteers were allocated to study phases (according to the treatment: aspartame, stannous-containing solution, or deionized water) following simple randomization procedures (computerized random numbers). The data analyst was kept blinded to the allocation of the volunteers and enamel blocks.

**Figure 1:**
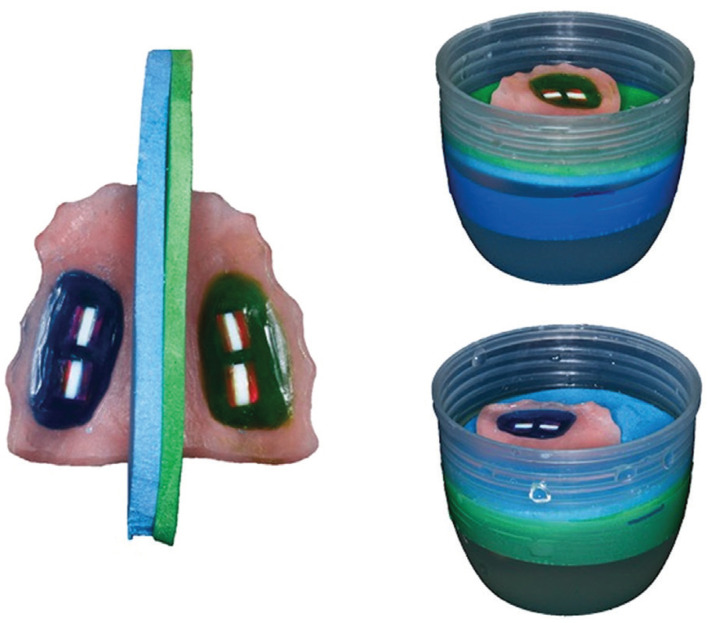
Intraoral appliance placed into an ethylene vinyl acetate dipositive allowing the immersion of half of the appliance in acid, while the other half floated without contacting the acid.

Each type of treatment was conducted in different study phases; erosion with citric and hydrochloric acids were performed in the same phase. A washout period of 7 days was established between the phases. The night before the beginning of each phase, the volunteers wore the intraoral appliances overnight (11.00 until 7.00) to allow formation of acquired enamel pellicle. Thereafter, volunteers wore the appliance for 5 working days from 7.45 to 18.00 (times could vary +/- 30 min) (11). The experimental procedure was as follows (all times +/- 15 min): at 7.45 the appliance was worn for pellicle formation; at 8.00 and 10.00 the treatment + erosive challenge was performed; at 12.00 the appliance was removed and stored in wet gauze during lunch; at 13.45 the appliance was worn for pellicle formation; at 14.00 and 16.00 the treatment + erosive challenge; and at 18.00 the appliance was removed and stored in wet gauze during night sleep. The participants were instructed not to eat while wearing the appliances; drinking water was allowed. Four times per day the volunteers rinsed the respective solutions (10 mL for 60 seconds) with the intraoral appliance *in situ*. Immediately after the mouthwash, the volunteers placed the appliance into an ethylene vinyl acetate dipositive that allows the immersion of half of the appliance in acid, while the other half floated without contacting the acid (Fig. 1) (12). Therefore, half of the appliance were immersed in 80 mL of 0.03M citric acid (natural pH 2.4) and the other half in 80 mL of 0.01M hydrochloric acid (adjusted with NaOH pH 2.3, to approximate that found in stomach acid) (13), both for 120 seconds without agitation and then washed in tap-water. A 2-hour period was planned for *in situ* exposure between challenges, during which the volunteers were asked not to remove the appliance (14,15).

-Final profilometric analysis

After the *in situ* phase, the nail varnish was removed and the profilometric analysis was performed at the same sites of the baseline measurements. Since the enamel samples could be precisely repositioned on the profilometer table, the respective baseline and final profiles could be matched. The graphs were superimposed and analyzed using a specific software program (MarSurf XCR 20, Göttingen, Germany). The vertical difference (average depth of the surface) between the baseline and final surface profiles were analyzed to quantify the enamel loss, reported as the mean of five graphs.

-Statistical analysis

Statistical analysis was performed with SigmaPlot version 12.3 (2011 Systat Software, Germany). The assumptions of equality of variances and normal distribution of errors were checked, since they were satisfied, two-way ANOVA and Tukey’s post hoc test were applied. The significance level was set at 5%.

## Results

Of the twenty-one volunteers, one was excluded for not following the *in situ* protocol properly. Table 1 contains the mean and the standard deviation for enamel loss of each studied groups. A significant difference was observed among treatments (*p*=0.000) and between type of acid (*p*=0.001). There was no interaction between type of acid and treatment (*p*=0.14). Hydrochloric acid promoted higher enamel loss than citric acid. Aspartame did not differ from negative control (deionized water) on enamel loss. Sn-containing solution significantly reduced enamel wear in comparison to the negative control and aspartame.

**Table 1:**
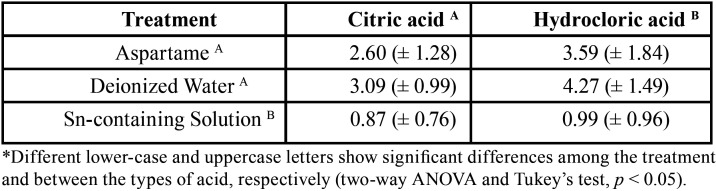
Means and standard deviations values of enamel loss (µm) of the studied groups.

## Discussion

The prevention of erosive tooth wear can be challenging since it is a multifactorial condition in which patient-related and nutritional factors are involved (4). Specific anti-erosive products could be useful when is not possible to intercept and reverse these causal factors. The present study evaluated the effect of an aspartame mouthwash against enamel erosive challenges by citric and hydrochloric acids, however this product was not able to prevent enamel loss. Therefore, the null hypothesis tested was rejected, due to a reduction in enamel loss by the stannous-solution.

Since 1981 to the present days, the Food and Drug Administration Agency (FDA) approves the use of aspartame and considers it safe as a general-purpose sweetener in food, being used as a non-nutritive sweetener in low-calorie products. Aspartame is a synthetic dipeptide formed by the reaction of L-aspartic acid with L-phenylalanine methyl ester, which can be released in contact with saliva (16). Amino acids, such as phenylalanine, may present potent antioxidant effect and may protect against inflammatory diseases (17). Phenylalanine has showed to attenuate ulcerogenic parameters and to improve the gastric hemorrhagic erosion in acid-irrigated stomachs of rats (18). Light cola – which contains aspartame – had demonstrated less erosive potential than regular cola (9,10). In the present study, the experimental aspartame mouthwash prior to acid challenge showed a reduction of only 16% of enamel loss in comparison with deionized water, without statistical differences. The stability of aspartame depends on pH and temperature; in aqueous solution, when the pH ranges between 4 and 5, the degradation is minimal. However, under extremely acidic conditions with pH below 4, aspartame can be degraded in phenylalanine, aspartic acid and methanol. Phenylalanine is considered to be an aromatic amino acid, having a carboxylic group and the amino group (19). Light cola soft drink has pH around 3 and thus, the protons of the acid might be possibly captured by the carboxylic (COO-) or aminic (NH2) groups of phenylalanine, diminishing deleterious action on the tooth enamel (9,10). In the present study, the pH of aspartame solution was 5.6, which may have impaired its anti-erosive effect. Different results might be obtained in solutions with lower pH (<4.0) which should be tested in future studies. Previous studies have found that at high concentrations, aspartic acid can bind to the N-methyl-D-aspartate receptor, causing an influx of calcium ions into brain cells (20). Also, the hydrogen peroxide and peroxyl radicals formed during the ingestion of aspartame seem to be involved in enhanced calcium mobilization in brain cells (19). Perhaps, in higher concentration, it could have some effect on tooth remineralization by enhancing calcium mobilization to acquired enamel pellicle, this hypothesis needs to be further evaluated.

The Sn-containing solution was the only treatment able to prevent enamel loss from citric and hydrochloric acids. This mouthwash solution contains 800 ppm of Sn2+ from stannous chloride, 125 ppm of F- from amine fluoride, and 375 ppm of F- from sodium fluoride and was adopted as positive control since it has previously showed preventive effect against tooth erosion (21). The stannous ions can form an acid-resistant layer on the surface of teeth increasing the substrate resistance. When it is associated with fluoride ions, the acquired enamel pellicle can be quantitatively and qualitatively modified forming a more stable and acid-resistant precipitates (21,22). Considering the pH of the Sn-solution (4.5), it could be interest to investigate in future studies its association with aspartame in order to improve the anti-erosive effect.

Considering the endogenous sources of acids, the mainly compound of gastric contend is hydrochloric acid, (23) being frequently adopted in experimental studies simulating this condition (24,25). Citric acid is one of the main acidic compound of exogenous sources, such as foodstuffs and drinks; (8) thus, it is frequently adopted in laboratorial studies to represent extrinsic causes of dental erosion (25,26). In the present study, citric and hydrochloric acid were adopted with the pH of 2.4 and 2.3 respectively, since many acidic drinks range at this level (8) and gastric juice has been simulated in this range of pH (6,25,26). It should be considered that in this experimental setup, acidic solutions of similar pH but different concentrations (0.01M for hydrochloric acid and 0.03M for citric acid) were used for characterization of erosive effect of intrinsic and extrinsic acids. Different acidic concentrations can impair the comparison with data, however previous study has found that concentration of the acids and the amount of titratable acid are of minor importance to determine the acidic erosive capacity when compared to pH and type of acid. Each erosive challenge was set in 120 seconds as in previous studies; (12,24) it was demonstrated that the pH on tooth surfaces stays low during this time after exposition to an acid until the salivary clearance. Hydrochloric acid is considered as a strong monovalent acid (pKa = −6.3 – HCl) and can dissolves and removes the mineral surface more quickly than weaker polyvalent acids, such as citric acid (pKa1 = 3.15; pKa2 = 4.77; pKa3 = 6.40). Thus, dental erosion from intrinsic acids seems to be more severe than from extrinsic acids (3). In the present study, hydrochloric acid promoted higher enamel loss than citric acid for all tested solution, which confirm this hypothesis.

Researches about preventive measures for erosive tooth wear should be ideally conducted *in vivo*. However due to the difficult to yield very precise intra-oral measurement of the progression of erosive wear *in vivo*; the present study was conducted *in situ/ ex vivo* (27). The intraoral appliance was exposed to the oral environment and the mouthwash was performed intra-orally, but the acid challenge was conducted extra-orally to avoid damage to the volunteer’s teeth (26). The enamel loss was measured by profilometry since it is considered a suitable method to measure the erosive wear thickness *in vitro* (27). The present study adopted bovine teeth, which is widely used in enamel erosion studies and it is easier to obtain in large quantities with a more uniform structure compared to human teeth (28). However, the presence of some chemical and structural differences should be taken into account when extrapolling the results to the clinical practice (28).

Based on the results of this study, the aspartame solution was not able to prevent the enamel loss when used before an erosive challenge independently of type of acid. Sn-solution promoted less enamel loss than deionized water and aspartame solution. Hydrochloric acid promoted higher enamel loss than citric acid. Further studies should be conducted evaluating the anti-erosive effect of an aspartame solution with lower pH.
